# Elevated Peritoneal Expression and Estrogen Regulation of Nociceptive Ion Channels in Endometriosis

**DOI:** 10.1210/jc.2014-2282

**Published:** 2014-07-16

**Authors:** Erin Greaves, Kelsey Grieve, Andrew W. Horne, Philippa T. K. Saunders

**Affiliations:** Medical Research Council Centre for Reproductive Health, The University of Edinburgh, Queens Medical Research Institute, Edinburgh EH16 4TJ, United Kingdom

## Abstract

**Context:**

Ovarian suppression is a common treatment for endometriosis-associated pelvic pain. Its exact mechanism of action is poorly understood, although it is assumed to reflect reduced production/action of estrogens.

**Objective:**

The objective of the study was to measure the expression of mRNAs encoded by nociceptive genes in the peritoneum of women with chronic pelvic pain (CPP) with or without endometriosis and to investigate whether estrogens alter nociceptive gene expression in human sensory neurons.

**Design:**

The study was performed using human tissue analysis and cell culture.

**Setting:**

The study was conducted at a university research institute.

**Patients:**

Peritoneal biopsies were obtained from women with CPP and endometriosis (n = 12), CPP and no endometriosis (n = 10), and no pain or endometriosis (n = 5). Endometriosis lesions were obtained from women with endometriosis (n = 18).

**Main Outcome Measures:**

mRNAs encoding ion channels (*P2RX3*, *SCN9A*, *SCN11A*, *TRPA1*, *TRPV1*) and the neurotransmitter *TAC1* were measured in human tissue samples and in human embryonic stem cell-derived sensory neurons treated with estrogens.

**Results:**

*TRPV1, TRPA1*, and *SCN11A* mRNAs were significantly higher in the peritoneum from women with endometriosis (*P* < .001, *P* < .01). *TRPV1, SCN9A*, and *TAC1* were elevated in endometriosis lesions (*P* < .05). P2RX3 mRNA was increased in the peritoneum of women with CPP, with and without endometriosis (*P* < .05). Incubation of sensory neurons with 17β-estradiol increased *TRPV1* mRNA (*P* < .01). The estrogen receptor-β-selective agonist 2,3-bis(4-hydroxy-phenyl)-propionitrile increased concentrations of *TRPV1, P2RX3, SCN9A*, and *TAC1* mRNAs.

**Conclusions:**

Estrogen-dependent expression of TRPV1 in sensory neurons may explain why ovarian suppression can reduce endometriosis-associated pain. Strategies directly targeting ion channels may offer an alternative option for the management of CPP.

Endometriosis is associated with debilitating chronic pelvic pain (CPP). It is an estrogen-dependent inflammatory disorder characterized by growth of endometrium (lesions) in ectopic locations, typically on the peritoneum and ovaries. Lesions become innervated by small diameter nerve fibers typical of afferent sensory innervation (1), and stimulation of these nerves by the inflammatory milieu within a lesion may be a direct cause of endometriosis-associated pain. Women with endometriosis often suffer from cyclical pain, and pain may occur in association with other conditions, such as irritable bowel syndrome and migraine (2, 3). Visceral, mechanical, and generalized hypersensitivity is extremely common in women with endometriosis (4), although there is no correlation between the extent of disease and reported pain scores (5). Stratton and Berkley (6) recently provided an overview of evidence for recruitment of nerves to endometriosis lesions, and they suggested that their stimulation and subsequent dialogue with the central nervous system may explain why women with endometriosis suffer from chronic pain.

Studies in patients with altered pain responses due to rare conditions as well as genetically modified mice (7) have identified key genes expressed by nerves that detect noxious stimuli (nociceptors). These include the TRP channel family (*TRPA1*, *TRPV1*), sodium voltage-gated ion channels (*SCN9A*, *SCN11A*) and purinergic X (*P2X*) receptors. An increase in the expression of neuropeptides, neurotrophins, and ion channels leading to an increased responsiveness of nociceptors has been suggested as an important contributor to development of hypersensitivity (8). For example, the TRPV1 channel (capsaicin receptor) has been implicated in development of hypersensitivity to heat associated with inflammation in chronic pain conditions (9). Recently studies have reported that TRPV1 immunoreactivity is increased in the peritoneum of women with CPP (10) and in lesions of women with endometriosis (11). The neuropeptide substance P (encoded by the *TAC1* gene) has been immunolocalized to endometriosis lesions (12), and TRPA1 has been implicated in thermal and mechanical hypersensitivity to pain (13). The purinergic X family of receptors are voltage-gated ion channels that open in response to ATP released from cells during inflammation and have also been associated with mechanical hyperalgesia (14). Sodium channels are also critical in producing an acute response to noxious stimuli; Nav1.7 (*SCN9A*) and Nav1.9 (*SCN11A*) are both reported to be increased in a range of inflammatory pain conditions (7). Notably, induction of a hypoestrogenic state often ameliorates CPP, and although the mechanisms responsible for this remain poorly understood, estrogens are reported to influence the sensitivity of peripheral sensory neurons and central neuronal activity in pain states (2).

In this study we measured the concentrations of mRNAs encoded by genes known to regulate nociception in tissue samples from women with CPP, some of whom were diagnosed with endometriosis. We also explored the impact of estrogens on their expression using a newly established in vitro model of human embryonic stem (ES) cell-derived sensory neurons.

## Materials and Methods

### Patients and recovery of tissue samples

The study was approved by the Lothian Research Ethics Committee (LREC 11/AL/0376). Peritoneal biopsies [∼1.0 × 0.5 cm size: stored in RNAlater (Applied Biosystems) at −20°C] were obtained from 3 groups of women (aged 18–45 y) at the time of surgery with informed consent: 1) women undergoing laparoscopic investigation for CPP with histological evidence of pelvic endometriosis (n = 12); 2) women undergoing laparoscopic investigation for CPP without evidence of endometriosis (n = 10); and 3) women undergoing laparoscopic sterilization without CPP and without evidence of endometriosis (n = 5). Endometriosis lesions were also collected from women with endometriosis (n = 18). Peritoneal biopsies from women without endometriosis were recovered from a site prone to endometriosis (pouch of Douglas) and in women with endometriosis the peritoneal biopsy was recovered from a site adjacent to the endometriosis lesion. None of the women had taken exogenous hormones for at least 3 months at the time of sampling.

### Differentiation of sensory neurons from human embryonic stem cells

Human ES cells, strain H9 (WiCell) were cultured on inactivated mouse embryonic fibroblasts maintained in Knockout DMEM/F12 supplemented with 20% knockout serum replacement (Gibco), 1 mM L-glutamine, 100 μM MEM nonessential amino acids, and 0.1 μM β-mercaptoethanol, with 6 ng/mL FGF-2 (R&D Systems). Differentiation of ES cells was induced using a cocktail of small molecule inhibitors as detailed by Chambers et al (15). RNA was extracted from cells at selected time points for the characterization and verification of phenotype. On day 22 the sensory neurons were trypsinized and reseeded into 12-well plates at equal densities (5 × 10^−5^ cells/well). On day 25, the cells were treated for 24 hours with 10^−8^ M 17β-estradiol (E2; Sigma), the estrogen receptor (ER)-α selective agonist 4,4′,4′-[4-propyl-(1H)-pyrazole-1,3,5-tryl] trisphenol (PPT), or the ERβ selective agonist 2,3-bis(4-hydroxy-phenyl)-propionitrile (DPN; Tocris) alone or in combination with the antiestrogen fulvestrant (ICI; 10^−7^ M; Tocris) dissolved in dimethylsulfoxide (DMSO).

### Immunocytochemistry

Expression of neurofilament protein was detected using neurofilament H chicken antineurofilament H (1:1000; Covance); nuclei were stained with 4′,6′-diamino-2-phenylindole and images captured on an Axiovert microscope (Carl Zeiss Inc).

### Calcium assay

Neurons were stimulated with 4 nM capsaicin (Sigma), and intracellular calcium was measured using a calcium indicator kit (BD Biosciences) with calcium flux captured using a NOVOstar microplate fluorometer (BMG Labtech).

### Quantitative real-time PCR

RNA was extracted from human tissues by homogenization in TRI reagent, chloroform phase separation and the lysates processed using an RNAeasy kit (QIAGEN). RNA was extracted from cells using RLT (lysis) buffer and an RNAeasy kit. Samples were deoxyribonuclease treated (QIAGEN) and concentration and purity assessed using a NanoDrop ND 1000. cDNA was synthesized using SuperScript VILO enzyme (Invitrogen) with 100 ng starting template in a 20-μL reaction. PCRs (15 μL) were performed using the Roche Universal Probe Library (Roche Applied Science) and Express quantitative PCR supermix (Invitrogen) with primers added at a concentration of 20 μM and thermal cycling performed on a 7900 Fast real-time PCR machine (Applied Biosystems) with *18S* selected as the reference gene because we have confirmed this gene is not altered by estrogenic treatments. cDNA was added at 1.5 μL per reaction and duplicate technical replicates performed.

Data were analyzed with RQ manager software (Applied Biosystems) using the δδCt method, and samples were normalized to either one laparoscopic sterilization sample (patients) or one vehicle control sample (cells). Primer sequences included the following: *TRPA1*, forward, 5′-tggacaccttcttcttgcatt-3′, reverse, 5′-tcatccatttcatgcagcac-3′; *TRPV1*, forward, 5′-agagtcacgctggcaacc-3′, reverse, 5′-ggcagagactctccatcacac-3′; *SCN9A*, forward, 5′-caacttttaagggatggacga-3′, reverse, 5′-tcatatttgggctgcttgtct-3′; *SCN11A*, forward, 5′-acctgagcctgaacaacagg-3′, reverse, 5′-tttgaactctctggctcgtg-3′; *P2RX3*, forward, 5′-ggcctttacttctgtgggagt-3′, reverse, 5′-aaacttcttggctttgtactggtc-3′; *TAC1*, forward, 5′-gcctcagcagttctttggat-3′, reverse, 5′-agcctttaacagggccactt-3′; *ER*α, forward, 5′-ttactgaccaacctggcaga-3′, reverse, 5′-atcatggagggtcaaatcca-3′; and *ER*β, forward, 5′-atcatggagggtcaaatcca-3′, reverse, 5′-tgggcattcagcatctcc-3′.

### Statistical analysis

Quantitative PCR (QPCR) data were analyzed using a one-way ANOVA and a Newman Keuls post hoc multiple comparison test. Statistics were generated using GraphPad Prism 6 software.

## Results

### mRNAs encoding nociceptive proteins exhibit altered expression patterns in the peritoneum of women with CPP

**Figure 1.**
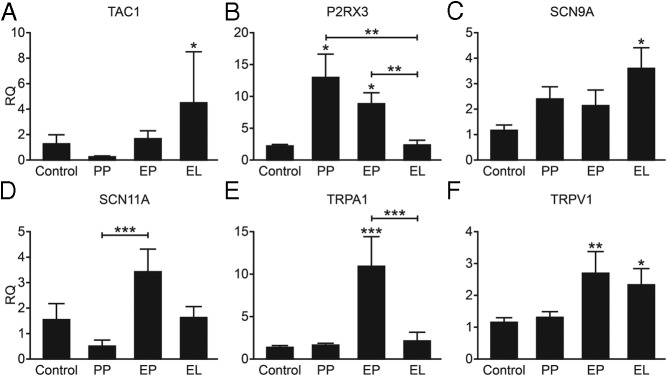
The neuropeptide *TAC1* and nociceptive ion channels are differentially expressed in CPP and in endometriosis. A–F, mRNA concentrations of the neuropeptide *TAC1* (A) and the nociceptive ion channels *P2RX3* (B), *SCN9A* (C), *SCN11A* (D), *TRPA1* (E), and *TRPV1* (F) were analyzed using QPCR. Concentrations of mRNAs were measured in the peritoneum of women with no pain (control; n = 5), compared with the peritoneum of women with CPP but no obvious underlying pathology (PP; n = 10), and the peritoneum (EP; n = 12) and peritoneal lesions (EL; n = 18) of women with confirmed endometriosis. RQ, relative quantification. Statistical analysis was performed using a one-way ANOVA combined with a Newman Keuls post hoc test. *, *P* < .05; **, *P* < .01; ***, *P* < .001. Asterisks denote statistical significance compared with control, and lines and asterisks denote significance between specific groups.

Measurement of mRNAs encoded by *TAC1* and nociceptive ion channels revealed differences between tissue samples; *TAC1* was elevated only in endometriosis lesions (Figure 1A; *P* < .05), and *P2RX3* was increased in the peritoneum of women with CPP, regardless of whether they were diagnosed with endometriosis compared with the peritoneum of healthy women (Figure 1B; *P* < .05). *SCN9A* (Nav1.7) was elevated in lesions from women with endometriosis (Figure 1C; *P* < .05), whereas *SCN11A* was significantly higher in the peritoneum of women with CPP and endometriosis compared with the peritoneum of women with CPP alone (Figure 1D; *P* < .001). mRNA encoded by *TRPA1* was significantly increased in the peritoneum of women with endometriosis compared with the peritoneum of healthy women and those with CPP alone (Figure 1E; *P* < .001). *TRPV1* was elevated in peritoneum (*P* < .01) and in lesions (*P* < .05) of women with endometriosis compared with the peritoneum of healthy women (Figure 1F).

### mRNA concentrations of nociceptive genes are regulated by estrogens in ES cell-derived sensory neurons

**Figure 2.**
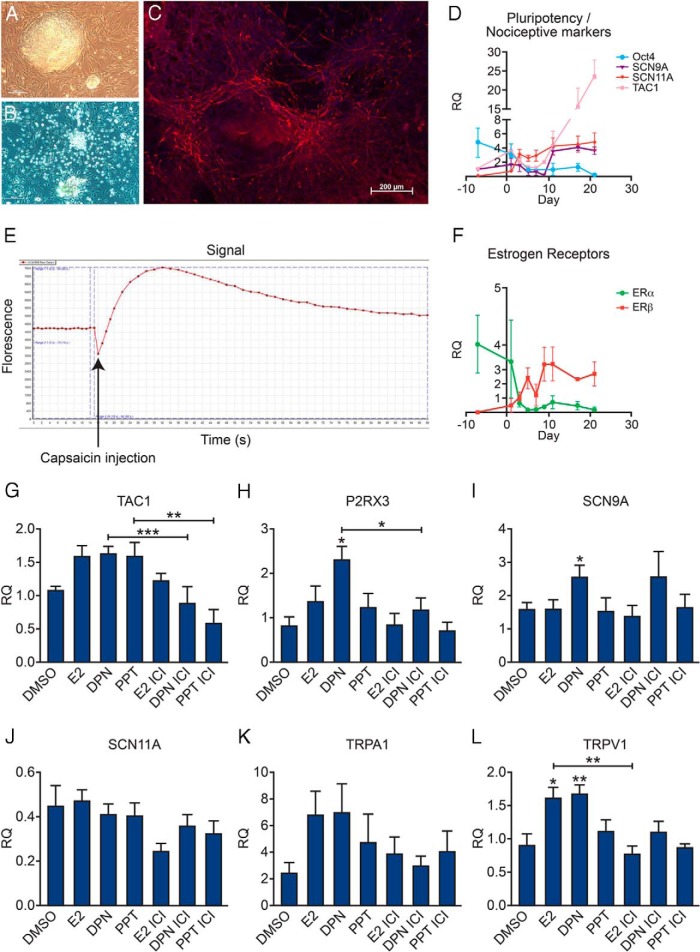
mRNA concentrations of nociceptive markers are regulated by estrogens in an in vitro model of human sensory neurons with a nociceptor-like phenotype derived from ES cells. A, Human ES cells were differentiated into sensory neurons with a nociceptor-like phenotype using combined small molecule inhibitors. Sensory neurons developed projections (B) and stained positively for neurofilament using immunocytochemistry (C). Scale bar, 200 μM. D, During the differentiation method, RNA was extracted from cells at different time points: pluripotent stem cells (d −7, n = 3), day 1 (n = 4), day 3 (n = 4), day 5 (n = 6), day 7 (n = 4), day 9 (n = 3), day 11 (n = 3), day 17 (n = 3), and day 21 (n = 3). QPCR analysis revealed that mRNA concentrations of the pluripotency marker octamer transcription factor-4 (*Oct4*) declined as the differentiation procedure progressed. The nociceptive markers *TAC1, SCN9A*, and *SCN11A* increased. E, Sensory neuron functionality was determined by stimulating cells with 4 nM capsaicin and recording calcium flux. F, During differentiation from ES cells to mature sensory neurons, *ER*α mRNAs declined, whereas *ER*β inclined. G–L, Mature sensory neurons were incubated with DMSO (vehicle), E2, DPN, or PPT, with or without ICI, and RNA was extracted after 24h. G–I, Using QPCR *TAC1* mRNAs were elevated by DPN and PPT compared to DPN + ICI and PPT + ICI. *P2RX3* and *SCN9A* mRNAs were elevated by DPN compared with vehicle control (DMSO). J and K, No significant differences were detected in *SCN11A* and *TRPA1*. L, *TRPV1* mRNAs were elevated by E2 and DPN. RQ, relative quantification. Statistical analysis was performed using a one-way ANOVA and Newman Keuls post hoc test. *, *P* < .05; **, *P* < .01; ***, *P* < .001. Asterisks denote statistical significance compared with vehicle control (DMSO), and lines and asterisks denote significance between specific treatment groups.

Sensory neurons immunopositive for neurofilament were generated in vitro (15) (Figure 2, A–C). Differentiation was accompanied by a reduction in octamer transcription factor-4 mRNA concentrations and an increase in *TAC1, SCN9A*, and *SCN11A* (Figure 2D), consistent with differentiation of sensory neurons with a nociceptor-like phenotype. When the differentiated cells were incubated with capsaicin (activates TRPV1), an intracellular calcium flux was detected (Figure 2E). The expression of ER subtypes changed during differentiation; *ER*α mRNA concentrations decreased and *ER*β mRNAs increased (Figure 2F). Stimulation of sensory neurons with the estrogen ligands E2, DPN, and PPT revealed ER-dependent regulation of nociceptive ion channels. *TAC1* and *P2RX3* mRNAs were increased after the incubation with DPN compared with cells incubated with DPN in the presence of ICI (Figure 2G; *P* < .001) or vehicle control (DMSO; Figure 2H; *P* < .05), respectively. *TAC1* was also elevated by PPT compared with PPT + ICI (Figure 2G; *P* < .01). Although *SCN9A* increased with DPN (*P* < .05), this was not blocked by the addition of ICI (Figure 2I), and the expression of *SCN11A* (Figure 2J) and *TRPA1* (Figure 2K) did not appear to be ER dependent. In contrast, *TRPV1* mRNA concentrations were elevated by both E2 (*P* < .05) and DPN (Figure 2I; *P* < .01), and this effect was abrogated by ICI.

## Discussion

We believe this is the first study to detect differences in the expression of mRNAs encoded by the ion channels *TRPA1*, *P2RX3*, *SCN9A*, and *SCN11A* in the peritoneum and lesions of women suffering from CPP, some of whom had endometriosis. We believe these insights may be indicative of sensitization of nerves present within the peritoneal lining of women with pain and therefore shed new light on the mechanisms responsible for development of chronic hypersensitivity.

In women with active endometriosis, we discovered that the concentrations of mRNAs encoded by *SCN11A*, *TRPA1*, and *TRPV1* were all significantly increased in the samples of peritoneum. We believe these novel findings are consistent with nociceptive changes taking place within the peritoneum of women with endometriosis, suggesting an increased sensitization of the peritoneum adjacent to the lesion. We detected significant increases in *TRPV1* mRNAs in both the peritoneum and in lesions, confirming and extending a previous study (11). Notably, *P2RX3* mRNAs were significantly elevated in the peritoneum of women with CPP, regardless of whether they had active endometriosis and would be consistent with a neuropathic component to idiopathic CPP.

To date, researchers have used rat or chick dorsal root ganglia (DRG) neurons as an in vitro model to investigate the function of sensory neurons. However, because DRG neurons represent a mixed population of somatosensory neurons, the development of a differentiation protocol allowing the generation of a population of human sensory neurons with a nociceptor-like phenotype (15) is particularly welcome. In the current study, we detected expression of estrogen receptor mRNAs in our ES cell-derived cultures. Treatment of cells with E2 or the ERβ agonist DPN induced a significant increase in *TRPV1* mRNAs that was blocked by the addition of the ER antagonist ICI. In addition, *P2RX3, SCN9A*, and *TAC1* mRNAs were all increased when cells were incubated with the ERβ agonist DPN, and *TAC1* was also increased by PPT. We have previously demonstrated DPN-dependent changes in gene expression in endometrial endothelial cells and have shown that these were mediated via specificity protein-1 tethered transcription (16). Bioinformatics revealed the presence of specificity protein-1 transcription factor binding sites in the promoter regions of *TRPV1*, *P2RX3*, *SCN9A*, and *TAC1* (Match; Biobase international.com). *TRPV1* mRNA was the only transcript that was increased in response to E2 treatment and abrogated by the antiestrogen ICI. Notably, bioinformatics detected several estrogen response elements in the promoter region of *TRPV1* and throughout the length of the transcript (Dragon estrogen response element locator version 6.0; National Institute of Health) that could facilitate direct binding of E2-activated ER receptor dimers. Further studies are required to validate these findings.

Estrogens are reported to have both pro- and antinociceptive properties. Mice exhibit hyperalgesia when estrogens are depleted after ovariectomy (17), and nociceptive responses are lower in ERβ knockout mice during the early stages of inflammation (18). The results of our study suggest estrogens may have a direct impact on nociception by up-regulating the expression of ion channels and would be consistent with reports that the expression of Trpv1 and P2x3 proteins are decreased in DRGs from mice with targeted deletions of *Esr2* and *Esr2* (19).

In summary, the results of this study indicate that therapies targeting P2RX3 may be useful in treating CPP in women with a diverse range of painful etiologies, whereas women with active endometriosis could benefit from treatments targeting TRPV1, TRPA1, SCN9A, or SCN11A. Studies using sensory neurons suggest regulation of TRPV1 by estrogens may provide an explanation for the reduction in pain experienced when ovarian steroids are suppressed and an opportunity for development of novel therapies.
